# Footprints in the scan: reducing the carbon footprint of diagnostic tools in urology

**DOI:** 10.1097/MOU.0000000000001196

**Published:** 2024-06-06

**Authors:** Alexandre Woernle, Caroline M. Moore, Clare Allen, Francesco Giganti

**Affiliations:** a Faculty of Medical Sciences; b Division of Surgery & Interventional Science, Faculty of Medical Sciences, University College London; c Department of Urology; d Department of Radiology, University College London Hospital NHS Foundation Trust, London, UK

**Keywords:** carbon footprint, imaging, radiology, sustainability, urology

## Abstract

**Purpose of review:**

There is an ever-growing focus on climate change and its impact on our society. With healthcare contributing a sizeable proportion of carbon emissions, the sector has a duty to address its environmental impact. We highlight the recent progress, current challenges, and future prospects for reducing the carbon footprint in diagnostic urology, specifically for imaging, without compromising patient care.

**Recent findings:**

The review is separated into four key areas of recent research: the design of a green radiology department, considering both infrastructural as well as behavioural changes that promote sustainability; individual scanners, where we provide an update on recent technological advancements and changes in behaviour that may enhance sustainable use; responsible resource allocation, where it is important to derive the maximal benefit for patients through the smallest use of resources; the recent research regarding single versus reusable urologic endoscopes as a case example.

**Summary:**

We offer an overview of the present sustainability landscape in diagnostic urology with the aim of encouraging additional research in areas where existing practices may be challenged. To protect the environment, attention is drawn to both more simple steps that can be taken as well as some more complex and expensive ones.

## INTRODUCTION

Climate change, a term which encompasses both global warming (a rise in Earth's average atmospheric temperature) and increases in the prevalence and magnitude of more extreme weather patterns, is at the forefront of discussion at the governmental, institutional, and individual level [1]. Over recent decades, there has been a growing awareness of its potential forecasted consequences and a heightened desire to tackle them. Worldwide consensus that climate change is human-driven, mainly through greenhouse gas production [2] is equally reassuring and daunting; while there are ample opportunities to mitigate the course and impact of climate change, they are only possible if timely efforts are made on a global scale [1].

In auditing an entity's contribution to climate change, it is crucial to consider the carbon footprint, a quantitative measure of the amount of greenhouse gases emitted because of a particular activity [3]. Logically, efforts to combat climate change involve minimizing carbon footprint. As a sector, healthcare contributes over 5% of carbon emissions worldwide – positioning it as high as the fifth largest polluter if it were ranked as a country [2]. Therefore, the medical field as a whole has a responsibility to attempt to minimize, where possible, its sizeable contribution to climate change. Promoting sustainability in medicine is crucial but patient safety and benefit should never be compromised. The foremost priority is to provide the most accurate and effective investigations or treatments, irrespective of their environmental impact.

We investigate the methods by which urology, as a specialty, can reduce its carbon footprint for diagnostic procedures. There is particular focus on the impact of imaging, said to account for 0.77% of emissions worldwide in computed tomography (CT) and magnetic resonance imaging (MRI) alone [4]. Bringing together research from both within as well as outside of the specialty, we identify four key settings within diagnostic urology where opportunities for improved sustainability exist. 

**Box 1:**
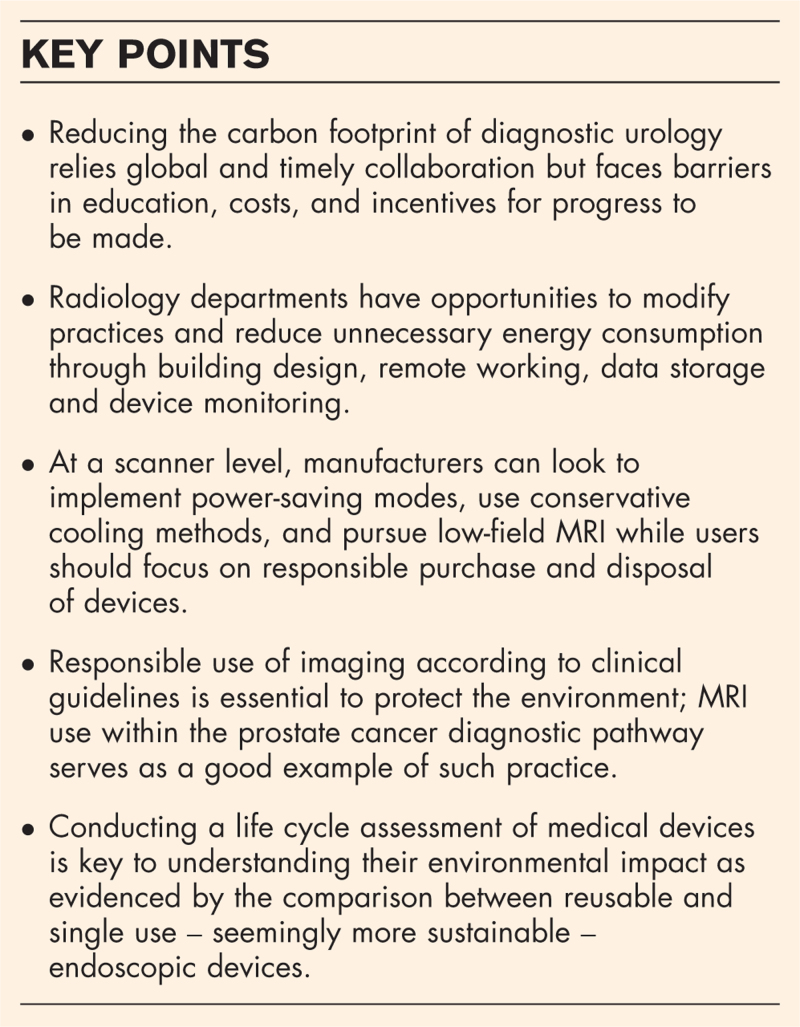
no caption available

## SETTING 1: DESIGNING A GREENER RADIOLOGY DEPARTMENT

To minimize energy losses to the surroundings, it is possible to store heat intelligently and reroute its path [5]. One centre has found that recovering heat from the MR scanners could be used to power more than three quarters of the building's heating [6]. In the layout of the radiology department, it may also be possible to link scanners by a cooling system with a view to minimizing expenditure [5]. Heat retention is not an aim unique to medicine; developments in other building designs may be transferable to hospitals.

Radiology has been at the forefront of medicine's efforts to go paperless. For example, the Picture Archiving and Communication System (PACS) has become the standard in radiology departments, effectively eradicating the practice of film printing. Teleradiology (i.e., remote reporting) minimizes travel emissions, improves flexibility and allows for accessibility and sharing of images [7]. Where possible, hospitals should encourage this practice. However, owing to the constant need for electricity to power a data centre, we must remember that data storage represents an astonishing proportion of CO_2_ emissions hence we must take a rational approach to this. This is still a work in progress and involves finding a solution for the length of time data should be stored that is a compromise between patients, hospitals, and the environment [8].

It is a common finding that radiology departments worldwide have a considerable number of devices that are left in a ‘on’ state (up to a third more between idle and off [9^▪^]) leading to unnecessary energy consumption and hence avoidable carbon footprint. There is anecdotal evidence that as many as half of machines are not switched off out of hours [10^▪▪^]. To reliably quantify such behaviour, audits have yielded somewhat shocking results. It has been reported that imaging departments can contribute an excess of 51.2 tons of greenhouse gases per year through not turning off PACS stations and computers, which is estimated to be roughly similar to the same quantity produced by 10 cars over the same time frame [11]. A recent publication which described the creation of a monitoring program for real-time energy consumption found a trend in decreased consumption when real time energy consumption results were available [10^▪▪^]. Efforts such as these should be encouraged, and their software shared where possible for the benefit of other hospitals. If convenient, remote control with automatic scheduling could be a further possibility to optimize consumption – this could act as a backup to manual switching on/off of machines when not possible or forgotten.

## SETTING 2: SCANNERS: HOW CAN WE OPTIMIZE THEM?

An issue of greater complexity arises from how we make scanners more energy efficient. As is the case for a growing number of electronic devices, the availability of a power-save mode is an attractive option – but one not always available. It has been quoted that a third of MRI energy consumption occurs when the device is completely switched off, owing to the presence of a cooling system [5]. If this cooling process can be cycled to eliminate periods of redundancy [9^▪^] without allowing for helium to boil-off [12^▪▪^], this could result in considerably less energy expenditure. Furthermore, much like for computers and workstations, if some scanners can enter a low power state automatically after a predetermined period, this would allow for automated energy savings, requiring no staff input [13].

The issue of scan quality is an important one to highlight here, which has been shown to be variable worldwide [14]. Ensuring adequate scan quality minimizes the need for repeat scans or further investigations which, on top of allowing for an accurate diagnosis, reduces carbon footprint downstream. Traditionally, low field strength (<1.5 T) MRI was not used owing to issues with diagnostic quality and scan time [15]. In recent times, the advent of artificial intelligence reconstruction has meant that more rapid and lower field sequences may also achieve diagnostic quality with quicker time spent scanning [12^▪▪^]. This is an emerging field and is not yet applicable to all areas [14]. It has been shown that new MRI scanners using lower field strengths could have clinical usefulness, representing an attractive possibility [16], as their reduced field strength consumes less energy [12^▪▪^] than their higher strength counterparts. Klein [6] correctly points out that as a permanent magnet, the production of such a scanner requires significantly more carbon in comparison to those which use a superconductor, but this seems to be offset after a use of as little as four months. Some of these devices may also be equipped with remote scanning features, allowing for a technologist to undertake a scan from another location [17]. If implemented correctly, this could lead to reduced travel time for both patients and staff and better healthcare availability.

Nevertheless, one must be wary that scanner performance is contingent on its age and quality reduces over time [18]. As a general shift towards a greener practice, embracing a circular mindset with respect to regular scanner update or replacement ensures minimization of waste and reduction in carbon footprint [2,12^▪▪^], as well as making sense from a financial perspective. This represents a shift away from the historical donation of medical devices to lower income areas. While it may be seen as promoting healthcare accessibility worldwide, donations rely on the ability to receive such equipment in the perfect setting, that is, with the necessary technicians and repair capability at destination [12^▪▪^,^19^]. It has been reported that some donations do not comply with the World Health Organization guidelines on the subject, a set of prerequisites to ensure effective assimilation, and thus may go directly to waste [20]. Owners of medical equipment have a responsibility to ensure the minimization of such waste while suppliers are responsible for ensuring adequate upgrade and repair options exist. Whenever feasible, buying from sources that have obtained sustainable certification is advised.

## SETTING 3: DIAGNOSTIC PATHWAYS

### The prostate cancer diagnostic pathway

One of the pivotal diagnostic tools for the diagnosis of prostate cancer is MRI [21], which is not only beneficial for patient outcomes, but also for the environment if performed adequately. For example, MRI prior to biopsy as opposed to simply biopsy, can lead to the avoidance (or deferral) of almost 30% of biopsies [22]. This equates to a reduction of 1.4 million kg of carbon dioxide equivalent pollution for every 100 000 patients [23^▪▪^]. Focusing on this field reveals a debate regarding the superiority of MRI without intravenous injection of contrast medium vs. multiparametric MRI for the detection of clinically significant prostate cancer. Considering that use of contrast contributes approximately 10% more carbon-based pollution than the former [23^▪▪^], a demonstration of noninferiority of MRI without intravenous injection of contrast medium may pave the way for a more sustainable practice. Trials, such as PRIME [24] are currently ongoing to determine if centres could safely transition to the greener alternative.

Opportunities for telemedicine also exist within this area of urology. Accelerated by the COVID-19 pandemic, virtual consultations have improved access and minimized travel time for patients [25]. Moreover, the introduction of ‘one-stop’ clinics where the MRI procedure and ensuing consultation are combined into one hospital visit [26], has been a measure that has improved overall efficiency. Attending one clinical encounter as opposed to two avoids unnecessary travel-related emissions [27] as a by-product of ensuring a more rapid time to diagnosis. In scrutinizing the execution of such a pathway, other than the changes to scanners themselves, we can facilitate the efficient workflow of patients through increased staffing and improved scheduling – minimizing the time where scanners are left on but not in use [2,5].

### Responsible use of imaging

It is also beneficial to think about the utility of certain types of imaging in specific scenarios – an unnecessary investigation leads to additional carbon emissions [4] and radiation exposure (if an X-ray, CT scan or nuclear imaging) [28]. The variation in energy consumption between modalities is immense: a CT exam consumes 8–14 times more energy than an ultrasound, while an MRI consumes approximately 3–5 times that of a CT [29]. Anecdotal evidence suggests that imaging overuse is an established phenomenon [30,31], but few have attempted to quantify it. In surveying the literature, Hendee *et al.* report an estimate that between 20% and 50% of imaging studies are unnecessary for patient outcomes [28]. Importantly, they caveat that this figure does not appreciate the importance of a negative scan in ruling out pathology and is thus an over-inflation. A study on the American College of Radiology (ACR) appropriateness criteria – a set of guidelines to ensure sensible use of imaging facilities – found that the choice of scan could have been swapped to another “usually appropriate” scan type 48% of the time [32], which raises the question as to whether there are opportunities for a greener choice to be made in certain situations. However, there is no stronger evidence on the safety and accuracy of these such replacements.

Regardless of the exact figures, inappropriate use of imaging puts a strain on healthcare systems as well as the environment. This encourages services to audit their imaging departments, as has been done in the past for MRI of the head and spine in two hospitals in the United States. Here, the authors found that lumbar MRI was often inappropriately ordered, prompting further research into current guidelines and physician attitudes [33]. An incorporation of sustainability into the ACR appropriateness criteria could be trialled, as reported by Arepally *et al.*[34]. It is also interesting to note that considerable variation exists in CT and MRI usage across the G20 group of countries [35^▪^]. Although this report appears to support the earlier concerns about excessive imaging, reaching definitive conclusions from this analysis is challenging without considering factors such as healthcare accessibility, disease prevalence, or a comparison of clinical guidelines across these nations.

## SETTING 4: ENDOSCOPY: SINGLE USE VS. REUSABLE

Introduced to minimize cross-contamination and improve reliability [36], disposable ‘single-use’ endoscopic devices have now become a feasible alternative to reusable ones [37]. From a sustainability perspective, conducting a life cycle assessment – which tracks a product's environmental footprint from “raw material acquisition…to waste management” [38] – of a medical device prevents potential oversight of its overall environmental impact. Simply put, the discussion about single-use versus reusable endoscopic instruments revolves around comparing the carbon footprint of manufacturing and disposing of a single-use device with that of the sterilization process for reusable instruments.

This issue has been investigated for cystoscopy where three of the four studies on the subject have tended to favour single use cystoscopes. Baboudjian *et al.*[37], Hogan *et al.*[39] and Jahrreiss *et al.*[40] found their single-use cystoscope to have a smaller carbon footprint than the reusable one, while Kemble *et al.*[41] reported the opposite. Close analysis of the results in the various studies revealed that these discrepancies were almost entirely due to differing energy demands of the sterilization method used [42], for example Baboudjian *et al.*[37] described a more energy intensive manual method – with Hogan *et al.*[39] and Jahrreiss *et al.*[40] showing similar demands – while Kemble *et al.*[41] described using two forms of automation with better energy efficiency. To our knowledge, only Davis *et al.*[43] have reported on the carbon footprint of ureteroscopy – which they found to be highly similar between both types of devices. If choosing a reusable device, the case load between maintenance and repairs are an important consideration [39] – especially since ureteroscopes are more susceptible to damage than cystoscopes [41].

This issue will always be contentious to a certain degree, as some of the data used in a life cycle assessment is gathered from estimates that may not be applicable to the device in question. Nevertheless, it is essential to highlight the importance of geographical context for these studies. Heavy energy consumption does not necessarily translate into a large carbon footprint - this depends on the source of energy [42]. In countries which have a high proportion of energy derived from renewable sources, it may be more sustainable to expend energy on sterilization of existing devices, instead of manufacturing additional instruments which lead to further waste. In addition, owing to the heterogeneity in disinfection methods and tube design, it is important for centres to conduct life cycle assessment of their own endoscopic practices to gauge whether they are adopting the most sustainable practice.

## CONCLUSION

The state of the environment is of growing concern worldwide. In the UK, in line with overwhelming public opinion, the National Health Service has outlined a plan to achieve net zero emissions by 2045 [44]. We applaud the increasing discussion and pressure to promote eco-friendly initiatives within the health sector. Research which challenges the sustainability of the status quo is of great encouragement to improve outcomes for patients whilst minimizing impact on the environment.

In highlighting just a few key areas for improvement, we acknowledge that this review only scratches the surface of the broader scope of urology's carbon footprint. There are several reviews which address changes that can be made from an interventional perspective, for example analysing the use of anaesthetic agents in operating theatres [45] or single use versus reusable catheterization [46].

Much of what can be done within healthcare from a sustainability perspective relies on optimizing use of preexisting resources. Developments of more energy efficient scanners in industry is both costly and laborious, and in many ways could be seen as the last piece of the jigsaw puzzle. We must first work towards streamlining pathways, promoting sensible use of imaging, encouraging conservative use of devices, buying and recycling sustainable equipment and building green departments.

However, as outlined by Brown *et al.*[2] earlier this year, we face three main challenges that may hinder our progress towards implementing these strategies: i) lack of education on the subject; ii) considerable financial cost, and iii) absence of direct incentives to make change. These obstacles may only be overcome through collaboration between governing bodies, industry players, institutions, and healthcare staff. For instance, we would like to draw attention to the world's first carbon-neutral radiology service in California [47] which will monitor consumption and investigate energy efficient solutions, amongst other measures to improve accessibility of care to the region.

The future in the field of sustainability in Urology and Radiology is a promising one but requires continued efforts to meet our objective of tackling climate change.

## Acknowledgements

*None.*

### Financial support and sponsorship

*None.*

### Conflicts of interest

*F.G. reports consulting fees from Lucida Medical Ltd outside of the submitted work.*

## REFERENCES AND RECOMMENDED READING

Papers of particular interest, published within the annual period of review, have been highlighted as:

▪ of special interest
▪▪ of outstanding interest

## References

[1] Romanello M Napoli CD Green C. The 2023 report of the Lancet Countdown on health and climate change: the imperative for a health-centred response in a world facing irreversible harms. Lancet 2023; 402:2346–2394. 37977174 10.1016/S0140-6736(23)01859-7 PMC7616810

[2] Brown M Schoen JH Gross J. Climate change and radiology: impetus for change and a toolkit for action. Radiology 2023; 307:e230229. 37070994 10.1148/radiol.230229

[3] Pandey D Agrawal M Pandey JS. Carbon footprint: current methods of estimation. Environ Monit Assess 2011; 178:135–160. 20848311 10.1007/s10661-010-1678-y

[4] Picano E Mangia C D’Andrea A. Climate change, carbon dioxide emissions, and medical imaging contribution. J Clin Med 2022; 12:215. 36615016 10.3390/jcm12010215 PMC9820937

[5] Heye T Knoerl R Wehrle T. The energy consumption of radiology: energy- and cost-saving opportunities for CT and MRI operation. Radiology 2020; 295:593–605. 32208096 10.1148/radiol.2020192084

[6] Klein HM. A new approach to the improvement of energy efficiency in radiology practices. Rofo 2023; 195:416–425. 36928520 10.1055/a-2021-7386

[7] Lu MT Tellis WM Fidelman N. Reducing the rate of repeat imaging: import of outside images to PACS. AJR Am J Roentgenol 2012; 198:628–634. 22358003 10.2214/ajr.11.6890

[8] Mariampillai J Rockall A Manuellian C. The green and sustainable radiology department. Radiologie (Heidelb) 2023; 63: (Suppl 2): 21–26. 37721584 10.1007/s00117-023-01189-6 PMC10689521

[9▪] Woolen SA Becker AE Martin AJ. Ecodesign and operational strategies to reduce the carbon footprint of MRI for energy cost savings. Radiology 2023; 307:e230441. 37097133 10.1148/radiol.230441

[10▪▪] Heye T Meyer MT Merkle EM Vosshenrich J. Turn it off! A simple method to save energy and CO(2) emissions in a hospital setting with focus on radiology by monitoring nonproductive energy-consuming devices. Radiology 2023; 307:e230162. 37070998 10.1148/radiol.230162

[11] McCarthy CJ Gerstenmaier JF AC ON. “EcoRadiology” – pulling the plug on wasted energy in the radiology department. Acad Radiol 2014; 21:1563–1566. 25175323 10.1016/j.acra.2014.07.010

[12▪▪] Chaban YV Vosshenrich J McKee H. Environmental sustainability and MRI: challenges, opportunities, and a call for action. J Magn Reson Imaging 2023; 59:1149–1167. 37694980 10.1002/jmri.28994 PMC11707703

[13] Büttner L Posch H Auer TA. Switching off for future – cost estimate and a simple approach to improving the ecological footprint of radiological departments. Eur J Radiol Open 2021; 8:100320. 33457469 10.1016/j.ejro.2020.100320 PMC7797527

[14] Woernle A Englman C Dickinson L. Picture perfect: the status of image quality in prostate MRI. J Magn Reson Imaging 2023; 59:1930–1952. 37804007 10.1002/jmri.29025

[15] Arnold TC Freeman CW Litt B Stein JM. Low-field MRI: clinical promise and challenges. J Magn Reson Imaging 2023; 57:25–44. 36120962 10.1002/jmri.28408 PMC9771987

[16] Hori M Hagiwara A Goto M. Low-field magnetic resonance imaging: its history and renaissance. Invest Radiol 2021; 56:669–679. 34292257 10.1097/RLI.0000000000000810 PMC8505165

[17] Hudson D Sahibbil JP. Remote scanning support in magnetic resonance imaging: friend or foe? Radiography (Lond) 2022; 28:739–745. 35410706 10.1016/j.radi.2022.03.010 PMC10073644

[18] Burn PR Freeman SJ Andreou A. A multicentre assessment of prostate MRI quality and compliance with UK and international standards. Clin Radiol 2019; 74:894.e19–894.e25. 10.1016/j.crad.2019.03.026 31296337

[19] Sumner C Ikuta I Garg T. Approaches to greening radiology. Acad Radiol 2023; 30:528–535. 36114076 10.1016/j.acra.2022.08.013

[20] McDonald S Fabbri A Parker L. Medical donations are not always free: an assessment of compliance of medicine and medical device donations with World Health Organization guidelines (2009–2017). Int Health 2019; 11:379–402. 30916303 10.1093/inthealth/ihz004

[21] Penzkofer T Tempany-Afdhal CM. Prostate cancer detection and diagnosis: the role of MR and its comparison with other diagnostic modalities—a radiologist's perspective. NMR Biomed 2014; 27:3–15. 24000133 10.1002/nbm.3002 PMC3851933

[22] Kasivisvanathan V Rannikko AS Borghi M. MRI-targeted or standard biopsy for prostate-cancer diagnosis. N Engl J Med 2018; 378:1767–1777. 29552975 10.1056/NEJMoa1801993 PMC9084630

[23▪▪] Leapman MS Thiel CL Gordon IO. Environmental impact of prostate magnetic resonance imaging and transrectal ultrasound guided prostate biopsy. Eur Urol 2023; 83:463–471. 36635108 10.1016/j.eururo.2022.12.008

[24] Asif A Nathan A Ng A. Comparing biparametric to multiparametric MRI in the diagnosis of clinically significant prostate cancer in biopsy-naive men (PRIME): a prospective, international, multicentre, noninferiority within-patient, diagnostic yield trial protocol. BMJ Open 2023; 13:e070280. 10.1136/bmjopen-2022-070280 PMC10083803 37019486

[25] Rodler S Ramacciotti LS Maas M. The impact of telemedicine in reducing the carbon footprint in healthcare: a systematic review and cumulative analysis of 68 million clinical consultations. Eur Urol Focus 2023; 9:873–887. 38036339 10.1016/j.euf.2023.11.013

[26] Eldred-Evans D Connor MJ Bertoncelli Tanaka M. The rapid assessment for prostate imaging and diagnosis (RAPID) prostate cancer diagnostic pathway. BJU Int 2023; 131:461–470. 36134435 10.1111/bju.15899

[27] Ramacciotti LS Kaneko M Eppler M. Editorial comment: environmental impact of prostate magnetic resonance imaging and transrectal ultrasound guided prostate biopsy. Int Braz J Urol 2023; 49:383–385. 37115182 10.1590/S1677-5538.IBJU.2023.03.02 PMC10335886

[28] Hendee WR Becker GJ Borgstede JP. Addressing overutilization in medical imaging. Radiology 2010; 257:240–245. 20736333 10.1148/radiol.10100063

[29] Merkle EM Bamberg F Vosshenrich J. The impact of modern imaging techniques on carbon footprints: relevance and outlook. Eur Urol Focus 2023; 9:891–893. 37758613 10.1016/j.euf.2023.09.009

[30] Maskell G. Why does demand for medical imaging keep rising? BMJ 2022; 379:o2614. 36323408 10.1136/bmj.o2614

[31] McAlister S McGain F Petersen M. The carbon footprint of hospital diagnostic imaging in Australia. Lancet Reg Health West Pac 2022; 24:100459. 35538935 10.1016/j.lanwpc.2022.100459 PMC9079346

[32] Alshqaqeeq F McGuire C Overcash M. Choosing radiology imaging modalities to meet patient needs with lower environmental impact. Resour Conserv Recycl 2020; 155:104657.

[33] Emery DJ Shojania KG Forster AJ. Overuse of magnetic resonance imaging. JAMA Intern Med 2013; 173:823–825. 23529302 10.1001/jamainternmed.2013.3804

[34] Arepally A Omary RA Vandenbergh MP. Scanning the planet: radiology's grand opportunity to address climate change. J Am Coll Radiol 2022; 19 (Pt B):217–219. 35033315 10.1016/j.jacr.2021.08.031

[35▪] Furlan L Di Francesco P Tobaldini E. The environmental cost of unwarranted variation in the use of magnetic resonance imaging and computed tomography scans. Eur J Intern Med 2023; 111:47–53. 36759306 10.1016/j.ejim.2023.01.016

[36] Pasquale L Maurano A Cengia G. Infection prevention in endoscopy practice: comparative evaluation of re-usable vs single-use endoscopic valves. Infect Prev Pract 2021; 3:100123. 34368741 10.1016/j.infpip.2021.100123 PMC8336158

[37] Baboudjian M Pradere B Martin N. Life cycle assessment of reusable and disposable cystoscopes: a path to greener urological procedures. Eur Urol Focus 2023; 9:681–687. 36543725 10.1016/j.euf.2022.12.006

[38] Finnveden G Hauschild MZ Ekvall T. Recent developments in life cycle assessment. J Environ Manage 2009; 91:1–21. 19716647 10.1016/j.jenvman.2009.06.018

[39] Hogan D Rauf H Kinnear N Hennessey DB. The carbon footprint of single-use flexible cystoscopes compared with reusable cystoscopes. J Endourol 2022; 36:1460–1464. 35607858 10.1089/end.2021.0891

[40] Jahrreiss V Sarrot P Davis NF Somani B. Environmental impact of flexible cystoscopy: a comparative analysis between carbon footprint of Isiris® single-use cystoscope and reusable flexible cystoscope and a systematic review of literature. J Endourol 2024; 38:386–394. 38185843 10.1089/end.2023.0274

[41] Kemble JP Winoker JS Patel SH. Environmental impact of single-use and reusable flexible cystoscopes. BJU Int 2023; 131:617–622. 36515438 10.1111/bju.15949

[42] Hogan D Hennessey DB Response to Rizan. The carbon footprint of single-use flexible cystoscopes compared with reusable cystoscopes—clarification of methods due to apparent misinterpretation. J Endourol 2023; 37:1145–1146. 36262041 10.1089/end.2022.0683

[43] Davis NF McGrath S Quinlan M. Carbon footprint in flexible ureteroscopy: a comparative study on the environmental impact of reusable and single-use ureteroscopes. J Endourol 2018; 32:214–217. 29373918 10.1089/end.2018.0001

[44] Sustainability and the NHS, Public Health and Social Care system – Ipsos Mori survey. Sustainable Development Unit for NHS England 2016.Delivering a ‘Net Zero’ National Health Service: NHS England, NHS Improvement; 2020. Available at: https://www.england.nhs.uk/greenernhs/wp-content/uploads/sites/51/2022/07/B1728-delivering-a-net-zero-nhs-july-2022.pdf.

[45] Pandit K Yodkhunnatham N Bagrodia A Monga M. Sustainability in urology: ideas for a greener future. Eur Urol Focus 2023; 9:894–896. 37748950 10.1016/j.euf.2023.09.006

[46] Kornberg Z Wu J Wilmot H. A leak in the system: addressing the environmental impact of urologic care. Eur Urol 2023; 84:260–262. 37225526 10.1016/j.eururo.2023.04.035

[47] Berthold J. Siemens Healthineers and UCSF Create First Carbon-Neutral Radiology Imaging Service; 2021. Available at: https://www.ucsf.edu/news/2021/11/421756/siemens-healthineers-and-ucsf-create-first-carbon-neutral-radiology-imaging#:∼:text=Siemens%20Healthineers%20and%20UCSF%20Create%20First%20Carbon%2DNeutral%20Radiology%20Imaging%20Service,-New%20Agreement%20Focuses&text=Siemens%20Healthineers%20and%20UC%20San,radiological%20imaging%20in%20Northern%20California.

